# Community-Based Physical Activity Interventions for Individuals with Moderate to Severe Traumatic Brain Injury: Scoping Review Protocol

**DOI:** 10.2196/24689

**Published:** 2021-01-13

**Authors:** Enrico Quilico, Bonnie Swaine, Christophe Alarie, Angela Colantonio

**Affiliations:** 1 Rehabilitation Sciences Institute University of Toronto Toronto, ON Canada; 2 Centre de recherche interdisciplinaire en réadaptation du Montréal métropolitain Montréal, QC Canada; 3 École de réadaptation Université de Montréal Montréal, QC Canada; 4 Department of Occupational Science and Occupational Therapy University of Toronto Toronto, ON Canada; 5 KITE-Toronto Rehabilitation Institute University Health Network Toronto, ON Canada

**Keywords:** traumatic brain injury, community, physical activity, exercise, sex, gender

## Abstract

**Background:**

Long-term physical, cognitive, and psychosocial problems resulting from moderate to severe traumatic brain injury (TBI) can prevent individuals from returning to preinjury lifestyles because of significant challenges with employment, leisure, and relationships. While physical activity (PA) is proposed as a cost-effective method to alleviate problems after moderate to severe TBI, there is no review to date that synthesizes the evidence for PA in the community-based context. Further, although sex- and gender-based considerations in research are considered requisite to good science, there is no review on PA and TBI that has included this explicit focus.

**Objective:**

The purpose of this review is to map and synthesize the current evidence identified through a systematic search of community-based PA interventions for individuals of all ages with moderate to severe TBI and provide an overview of that evidence by asking the following research questions: (1) what are the characteristics of community-based PA programs for individuals with moderate to severe TBI, (2) what are the reported health-related outcomes and measurement tools used to evaluate them, and (3) what considerations have been given to sex and/or gender?

**Methods:**

Searches will be conducted of six academic databases for peer-reviewed articles. Two reviewers will independently screen the articles for inclusion and extract data for the analysis. The extracted data will be coded according to the Consensus on Exercise Reporting Template checklist and the Template for Intervention Description and Replication checklist to provide sufficient detail for replication.

**Results:**

The abstract screening was completed by two reviewers and the extracted data were analyzed. A qualitative synthesis and description of community-based PA interventions for individuals with moderate to severe TBI will be provided.

**Conclusions:**

This scoping review will generate new knowledge from published and publicly available literature. Dissemination of the results will include activities related to knowledge transfer for community-based PA after moderate to severe TBI for future research and practice. Evidence-based recommendations, future directions, potential limitations, use of online/digital components, and the possible need for a systematic review will be discussed as well.

**International Registered Report Identifier (IRRID):**

DERR1-10.2196/24689

## Introduction

### Background

Traumatic brain injury (TBI) is a leading cause of long-term disability around the world [1], and the physical, cognitive, and psychosocial consequences resulting from moderate to severe cases of TBI frequently lead to a significant reduction in employment, interpersonal relationships, and leisure activities after injury [2]. Moderate to severe TBI is considered to be a “chronic disease process” because of the progressive nature of associated problems that result in high economic, personal, and social costs that extend over a lifetime [3]. Further evidence of this chronic disease process is demonstrated through a risk of social failure with higher rates of unemployment [4], homelessness [5], and incarceration [6]. The abundance of long-term social and health problems after moderate to severe TBI therefore emphasize a need for interventions to address management in the chronic recovery time period (eg, >2 years postinjury), when individuals typically live in the community setting and may no longer be supported by health professionals.

### Physical Activity (PA) After TBI

PA is recommended as a nonstigmatizing approach to improve recovery after TBI [7], and positive PA habits are shown to positively influence community integration, mood, and quality of life [8]. In this context, PA is shown to reduce depressive symptoms [9] and positively affect cognitive functioning [10] after TBI, especially in the chronic phases of recovery [11]. In addition, and relevant to the chronic recovery time period, a Cochrane review about cardiorespiratory training demonstrated that PA positively affects fitness after TBI and is acceptable for individuals to perform without adverse events [12].

That same Cochrane review reported that although cardiorespiratory training is effective at increasing cardiorespiratory fitness for adults with moderate to severe TBI, the clinical value of including fitness training after injury for the improvement of secondary health-related outcomes could not be assessed because of a lack of available data [12]. A number of studies in that review were excluded based on the types of interventions (nonrandomized control trials), as well as the necessity for interventions to focus specifically on cardiorespiratory fitness according to frequency, intensity, time, and type of training. Thus, it is plausible that other modalities of PA intervention (ie, walking, Qigong, circuit training) that do not specifically target cardiorespiratory fitness may have contributed to the enhancement of other health-related outcomes, but they were not included. Since then, more recent PA studies for adults with moderate to severe TBI have reported promising health improvements with weight management [13], mood, social participation, and recovery after TBI [14,15], as well as independent exercise performed in community fitness centers with minimal guidance [16].

In addition, identifying sex- and gender-based considerations may be relevant to the use of PA for health-related outcomes among adults with moderate to severe TBI. For example, men and women with TBI may experience exercise differently based on varying recovery outcomes, social behaviors, and motivation to participate in community-based physical therapy [17,18]. This information was lacking from the Cochrane review cited above. Thus, providing a qualitative description and synthesis of the characteristics of PA interventions, their reported health-related outcomes, and the measurement tools used to evaluate them, as well as considerations provided for sex and/or gender, is warranted.

### Objectives

The purpose of this review is to map and synthesize the current evidence identified through a systematic search of community-based PA interventions for individuals with moderate to severe TBI and provide an overview of that evidence by asking the following research questions: (1) what are the characteristics of community-based PA programs for individuals with moderate to severe TBI?; (2) what are the reported health-related outcomes and measurement tools used to evaluate them?; and (3) what considerations have been given to sex and/or gender? For the purposes of this review, definitions of “PA,” “intervention,” and “community-based” are provided below. A relevant and timely summary of these findings may assist researchers and community-based organizations (ie, fitness centers, TBI and/or disability associations) with the design and implementation of PA programs for individuals with moderate to severe TBI, as well as the appropriate outcome measurement tools for their evaluation.

## Methods

### Identifying Relevant Studies

This protocol was prepared according to the checklist of PRISMA-ScR (Preferred Reporting Items for Systematic Reviews and Meta-Analyses Protocols Extension for Scoping Reviews [19]; Multimedia Appendix 1), as well as the step-by-step guidelines of Arksey and O’Malley [20], with enhancements provided by Levac et al [21].

#### Search Strategy

Searches will be limited to human studies written in English or French because of the language proficiency of the research team and the scope of this review. Searches will be developed and conducted by an information specialist at University Health Network (UHN)–Toronto Rehabilitation Institute with consultation and feedback from the research team, who have extensive content and methodological expertise in the area of rehabilitation and TBI. A detailed description of the search strategy for this scoping review is provided in Multimedia Appendix 2.

#### Databases

Searches will be conducted in MEDLINE(R) (in Ovid, including Epub Ahead of Print, In-Process & Other Non-Indexed Citations, and Ovid MEDLINE[R] Daily), Embase (in Ovid, including Embase Classic), Cochrane Central Register of Controlled Trials (in Ovid, CENTRAL), CINAHL (EBSCOhost), SportDISCUS (EBSCOhost), and PeDRO.

### Selection Criteria

#### Types of Participants

Studies including people of any age, sex, or gender with moderate to severe TBI living in the community will be included. Moderate to severe TBI includes nonpenetrating head injuries caused by external forces that are assessed to be between 3 and 12 on the Glasgow Coma Scale to reflect individuals who live with the greatest number of TBI-related impairments [22]. Penetrating head injuries or anoxic brain injuries (ie, caused by stroke) will be excluded.

#### Types of Studies

All studies of community-based PA interventions will be included. No restrictions will be placed on the type of study design or methods used. The full-text article or report of the study must be included; therefore, reports of research only available in abstract form will be excluded. Reviews will not be included. However, review references will be carefully hand-searched by the lead reviewer (EQ) to ensure that relevant studies about community-based PA programs are identified. Gray literature, including theses/dissertations, conference proceedings, policies, reports, protocols, and book reviews will be excluded in order to reflect high-quality empirical research about PA interventions that focus on health-related outcomes.

#### Types of PA

Studies that include any type of PA intervention provided in a community setting—including home-based, community center–based, gym-based, outdoor-based, and park- and recreation center–based interventions—will be included. Leisure interventions that do not include PA, exercise, or sport will be excluded (eg, arts and crafts, music). For the purposes of this review, the operational definition of PA is any bodily movement produced by skeletal muscles that results in energy expenditure and positively correlates with physical fitness. The operational definition of exercise is PA that is planned, structured, and repetitive, with an objective to improve or maintain physical fitness components. Finally, the operational definition of sport is further defined as being a form of leisure-time PA [23]. For the purposes of this review, fitness is defined as the state of being in good health, intervention is defined as the action or process of intervening for the purpose of improving health, and community-based is defined as an intervention provided in the community rather than in hospitals or institutions [24].

#### Types of Outcomes

The World Health Organization’s International Classification of Functioning, Disability, and Health (ICF) [25] will be used to establish the criteria for health-related outcomes relating to body functions and structures, activities, participation, and environmental factors. Reported perceptions about the quality of PA interventions (ie, implementation and adherence), as well as the barriers, facilitators, and environmental factors (eg, setting and accessibility) related to intervention delivery, will be included as well.

#### Screening

Electronic search results will be downloaded into EndNote X9 [26] and Covidence [27] software. EndNote is a management software that will facilitate backup reference information for each one of the identified studies. Covidence is a web-based software platform that streamlines the production of systematic/scoping reviews and will assist with the removal of duplicates and the screening process. Two of the authors (EQ and CA) will independently screen all titles and abstracts for eligibility according to the inclusion/exclusion criteria. To support the iterative process suggested by Levac et al [21], the reviewers will meet at the beginning, midpoint, and final stages of the abstract review process. Discrepancies will be resolved by mutual discussion with a third senior author (AC). For studies with insufficient information in the title and abstract, the full text will be retrieved to make a decision. A full-text review will be carried out for studies marked for possible inclusion. During full-text review, if both EQ and CA determine a study does not meet the criteria, it will be excluded. Any disagreements at this stage will be resolved through discussions with AC. The reasons for excluding a full-text study will be documented.

### Data Extraction

A Microsoft Excel data extraction form developed by the authors will be used to extract data about the PA intervention characteristics, health-related outcomes, and measurement tools, as well as any relevant information about sex- and gender-based considerations. Data about the intervention characteristics will be categorized according to the 16 items of the Consensus on Exercise Reporting Template (CERT) checklist [28] and the 12 items of the Template for Intervention Description and Replication (TIDieR) checklist [29]. This information will include characteristics such as the type of equipment used, whether the intervention was individual- or group-based, a description of progressions, home-based components, nonexercise components, adverse events, and setting. Additional information about the authors and study design, as well as participants’ gender, age, ethnicity, injury severity, key conclusions, and any digital/online components, will be recorded. Two authors (EQ and CA) will independently extract data from the first 5 to 10 included studies using the charting form and will meet with the senior author (AC) to determine if the approach is consistent with the research question.

### Data Analysis

Descriptive data on intervention characteristics, health-related outcomes, evaluation tools, and sex- and gender-based considerations will be extracted for a summary analysis and organized according to the respective ICF domains. Similarly, qualitative data on intervention experiences, evaluations, and barriers and facilitators to PA participation will be extracted for a qualitative summary analysis. Intervention characteristics about feasibility and acceptability will also be extracted to provide a synthesis (ie, summary matrix) of best practices for the use of PA in the community after moderate to severe TBI with regard to frequency, intensity, time, and type of PA according to sex and/or gender. Where possible, explicit details of any subgroup considerations based on PA types, contexts, time points, and participant demographics (ie, age, sex/gender, ethnicity, and injury severity) will be included. The findings about the characteristics of community-based PA interventions that improve health-related outcomes for individuals with moderate to severe TBI, as well as respective evidence-based recommendations, will be presented in relation to future research and practice.

## Results

Progress to date includes searches that were conducted on January 10, 2019, and updated on November 22, 2019, and October 23, 2020. Search strategies included the use of text words and subject headings (eg, Medical Subject Headings [MeSH], Emtree) related to TBI, exercise, and community settings. The search strategy was first developed in MEDLINE and subsequently translated to other databases. As of November 2020, abstract screening was completed and the extracted data were analyzed.

## Discussion

### Principal Findings

The aim of this review is to map and synthesize the evidence about community-based PA interventions for adults with moderate to severe TBI, their respective health-related outcomes, and any sex- and gender-based considerations. The results of this review are expected to inform a practical summary matrix of common types of PA interventions (eg, fitness, leisure-time PA, sport) and their health-related outcomes, as well as the feasibility of running them in the community under the current circumstances with COVID-19 and the necessity for digital/online components that respect physical distancing guidelines. In addition, given the relatively new and emerging understanding about the effect of sex and gender on recovery from TBI, the study will provide a novel review of sex- and gender-based considerations for PA after TBI. The findings from this review are expected to be published and disseminated through academic journals and conferences. It is anticipated that the findings will be relevant and useful for researchers and community organizations for the development, implementation, and evaluation of PA interventions that are designed to assist with the management of TBI-related sequelae in the community.

### Limitations

The main limitation of this review is related to publication bias, as it will exclude gray literature, such as theses/dissertations, conference proceedings, policies, reports, protocols, and book reviews. Thus, it is possible that omitting gray literature may affect the results of this review. A second limitation is related to the potentially wide range of PA intervention types and reported health-related outcomes leading to heterogeneity of studies to summarize. However, this review will still maintain an accurate and qualitative summary of the use of PA to improve health-related outcomes after moderate to severe TBI. Finally, only English- and French-language studies will be considered because of the language proficiency of the research team, so relevant data published in other languages may not be included.

### Comparisons with Prior Work

Consultation with stakeholders may provide opportunities for further insight and enhance knowledge transfer [30]. Therefore, in order to further disseminate the practical use of these findings, and as part of a larger grant that supports the exploration, development, and evaluation of a community-based PA program for adults with TBI, a consultation will be carried out with a multidisciplinary team of researchers and community organization representatives from both the fitness industry and the TBI community. The purpose of the consultation will be to share the summary of results from the review to generate future directions associated with the project and specific opportunities for knowledge transfer with experts in the field.

### Conclusion

This scoping review will generate new knowledge from published and publicly available literature. Research ethics board approval is therefore unnecessary. Dissemination of the results will include activities related to knowledge transfer for community-based PA after moderate to severe TBI for future research and practice. For example, evidence-based recommendations, future directions, potential limitations, and the possible need for conducting a systematic review will be discussed.

## Appendix

Multimedia Appendix 1 PRISMA-ScR (Preferred Reporting Items for Systematic Reviews and Meta-Analyses Extension for Scoping Reviews) checklist.

Multimedia Appendix 2 Search strategy.

## Abbreviations

CERT: Consensus on Exercise Reporting Template
ICF: International Classification of Functioning, Disability, and Health
MeSH: Medical Subject Headings
PA: physical activity
PRISMA-ScR: Preferred Reporting Items for Systematic Reviews and Meta-Analyses Extension for Scoping Reviews
TBI: traumatic brain injury
TIDieR: Template for Intervention Description and Replication
UHN: University Health Network
