# Supplementary material for: Community-Based Physical Activity Interventions for Individuals with Moderate to Severe Traumatic Brain Injury: Scoping Review Protocol
Source: JMIR Res Protoc. 2021 Jan 13;10(1):e24689. doi: 10.2196/24689 (PMC7840288; doi:10.2196/24689)
Supplement: Multimedia Appendix 2 [file resprot_v10i1e24689_app2.docx]

**Multimedia Appendix 2: Search Strategy**

**Search Narrative**

This strategy was first developed in Medline and was subsequently translated to other databases. It uses a number of concepts:

Concept A: lines 1 to 13 = Traumatic Brain Injury

Concept B: lines 15 to 52 = Exercise or physical activity

Concept C: lines 54 to 62 = Community based setting

The search strategy for this review sought to identify papers that discussed community-based exercise programs for individuals having experienced a traumatic brain injury. The search conducted is: A AND B AND C. This strategy was used in all searched databases, with the exception of PeDRO, where the B concept was omitted because of the scope of the database. Searches were limited to English and French language publications, and animal studies were excluded when possible. In other databases, additional limits and search fields have been used when applicable such as to exclude conference proceedings.

**Medline (Ovid) Search Strategy**

1 exp Brain Injuries/

2 exp Brain Injuries, Traumatic/

3 exp Brain Concussion/

4 Craniocerebral Trauma/

5 Head Injuries, Penetrating/

6 exp Head Injuries, Closed/

7 tbi*2.tw,kf.

8 mtbi*2.tw,kf.

9 wrTBI*2.tw,kf.

10 concuss*.tw,kf.

11 postconcuss*.tw,kf.

12 ((brain* or head* or cerebr* or crani* or skull* or intracran*) adj2 (injur* or trauma* or damag* or wound* or swell* or oedema* or edema* or fracture* or contusion* or pressur*)).tw,kf. (158489)

13 ((brain* or cerebr* or intracerebr* or crani* or intracran* or head* or subdural* or epidural* or extradural*) adj (haematoma* or hematoma* or hemorrhag* or haemorrhag* or bleed*)).tw,kf. (47589)

14 or/1-13

15 exp Exercise/

16 exp Exercise Therapy/

17 exp Exercise Movement Techniques/

18 Physical Fitness/

19 exp "Physical Education and Training"/

20 exp Sports/

21 Recreation/

22 (exercise* or exercising).tw,kf.

23 (qi gong or qigong or gi gong or gigong).tw,kf.

24 ((tai adj ji) or ((tai or thai) adj chi) or taiji or taijiquan or taichi).tw,kf.

25 walking.tw,kf.

26 yoga.tw,kf.

27 (physical* adj (fit or fitness or condition* or education or training or mobility or activit* or exertion or effort or program* or therap*)).tw,kf.

28 gymnastic?.tw,kf.

29 calisthenic?.tw,kf.

30 aerobic?.tw,kf.

31 danc*.tw,kf.

32 (jumping or hopping).tw,kf.

33 (running or jogging).tw,kf.

34 muscle strengthening.tw,kf.

35 ((strength or resistance) adj training).tw,kf.

36 (fitness adj training).tw,kf.

37 ((weight? adj2 lifting) or weightlifting or power lifting or weight training).tw,kf.

38 pilates.tw,kf.

39 stretching.tw,kf.

40 plyometric*.tw,kf.

41 (cardio* adj (conditioning or training)).tw,kf.

42 ((physical or motion or movement or recreation or activity) adj therap*).tw,kf.

43 isometric training.tw,kf.

44 climbing.tw,kf.

45 cycling.tw,kf.

46 (swim or swimming).tw,kf.

47 (training adj (course* or program*)).tw,kf.

48 kinesi?therap*.tw,kf.

49 sport?.tw,kf.

50 ((multimodal or multi-modal or multicomponent or multi-component) adj training).tw,kf.

51 balance training.tw,kf.

52 ((lifestyle or physical) adj3 (course* or program* or module?)).tw,kf.

53 or/15-52

54 Community Health Services/

55 communit*.af.

56 (fitness adj2 (center? or centre?)).tw,kf.

57 (wellness adj2 (center? or centre?)).tw,kf.

58 (health adj2 club?).tw,kf.

59 (sport* adj2 club?).tw,kf.

60 (leisure adj2 (center? or centre?)).tw,kf.

61 gymnasium?.tw,kf.

62 free living.tw,kf.

63 or/54-62

64 14 and 53 and 63

65 64 not (exp animals/ not exp humans/)

66 65 and (eng or fre).lg.
